# Evolution of Antimicrobial Resistance of Uropathogen *Escherichia coli* in an Emergency Hospital from Bucharest

**DOI:** 10.3390/microorganisms14102199

**Published:** 2026-10-01

**Authors:** Codruța-Georgiana Carp, Constantin Florin Ghiațău, Gabriel Adrian Popescu

**Affiliations:** 1 University of Medicine and Pharmacy “Dr. Carol Davila”, 020021 Bucharest, Romania; codruta-georgiana.carp@drd.umfcd.ro; 2 Central Military Emergency University Hospital “Dr. Carol Davila”, 010825 Bucharest, Romania; 3 “Prof. Dr. Matei Balș” National Institute of Infectious Diseases, 021105 Bucharest, Romania

**Keywords:** *Escherichia coli*, antimicrobial resistance, urinary tract infection, urine culture

## Abstract

*Escherichia coli* is the leading uropathogen and a sentinel organism for antimicrobial resistance (AMR), yet urine culture over-ordering is an increasingly recognized, modifiable driver of unnecessary antimicrobial use. We evaluated both urinary *E. coli* resistance and urine culture utilization over a five-year period encompassing the COVID-19 pandemic in a Romanian tertiary hospital. In this retrospective single-center study (Central Military University Emergency Hospital “Dr. Carol Davila”, Bucharest; January 2019–December 2023), adult inpatient urine culture utilization was expressed per 1000 occupied bed-days (OBD) and modelled using quasi-Poisson and cluster-robust negative-binomial regression, while resistance was assessed from the first isolate per patient using linear probability and logistic regression. Among 95,840 cultures (16.3% positive), 7957 yielded *E. coli* (56.5% of positives; 76.5% female; mean age 64.1 years). OBD-adjusted utilization rose by 4.4% annually (IRR 1.044; *p* < 0.001), driven mainly by medical wards. Resistance was largely stable, with significant declines for nitrofurantoin and amoxicillin–clavulanic acid; aminopenicillins, trimethoprim–sulfamethoxazole, and fluoroquinolones exceeded the 20% empirical-use threshold, whereas nitrofurantoin and fosfomycin remained low. Rising bed-day-normalized utilization identifies medical wards as priority diagnostic-stewardship targets, while stable resistance supports nitrofurantoin and fosfomycin as preferred empirical agents, arguing for combined stewardship and activity-adjusted surveillance.

## 1. Introduction

Antimicrobial resistance has emerged as one of the most significant challenges to public health, compromising the effectiveness of antibiotics and increasing morbidity, mortality, and healthcare costs worldwide [1]. Among the bacterial pathogens associated with antimicrobial resistance, *Escherichia coli* occupies a central role due to its ubiquity in both community and hospital settings and its ability to acquire and disseminate resistance determinants rapidly [2]. *E. coli* is a common commensal organism of the human gastrointestinal tract; however, certain strains are responsible for a wide range of infections, including urinary tract infections (UTIs), bloodstream infections, neonatal meningitis, and gastrointestinal diseases [3]. According to the European Antimicrobial Resistance Surveillance Network (EARS-Net) data, across the European Union (EU) and the European Economic Area (EEA), in 2022, *E. coli* was responsible for 39.2% of all invasive infections caused by the eight bacterial species monitored by this network: *Streptococcus pneumoniae*, *E. coli*, *Klebsiella pneumoniae*, *Acinetobacter baumannii*, *Pseudomonas aeruginosa*, *Staphylococcus aureus*, *Enterococcus faecalis*, and *Enterococcus faecium* [4].

UTIs represent one of the most frequent bacterial infections encountered in both outpatient and hospital settings, with *E. coli* accounting for approximately 70–90% of community-acquired UTIs [5]. The initial empirical treatment of community UTIs traditionally includes antibiotics such as nitrofurantoin, trimethoprim-sulfamethoxazole, fosfomycin, fluoroquinolones, and β-lactam antibiotics, selected according to local antimicrobial susceptibility patterns and international clinical guidelines [6]. Early initiation of effective empirical therapy is essential to reduce symptom duration and limit the progression to severe infections such as pyelonephritis or urosepsis, which may in turn lead to complications including bacteremia, sepsis, multiple organ dysfunction, shock, and/or acute renal failure.

However, the increasing prevalence of antimicrobial-resistant *E. coli* strains has significantly complicated the management of UTIs worldwide, albeit to varying degrees and with differing resistance patterns across geographical regions [7]. Rising resistance rates to commonly prescribed antibiotics, particularly fluoroquinolones, aminopenicillins, and trimethoprim–sulfamethoxazole, have reduced the effectiveness of standard empirical regimens and increased the risk of therapeutic failure [8]. In addition, the emergence and dissemination of extended-spectrum β-lactamase (ESBL)-producing *E. coli* strains have further limited oral treatment options and frequently necessitated the use of agents active against multidrug-resistant Gram-negative bacilli, such as carbapenems and requiring intravenous administration [9]. These changes have important clinical and economic consequences, including prolonged hospitalization, increased healthcare costs, recurrent infections, and higher morbidity rates [10].

The COVID-19 pandemic has further influenced the epidemiology of antimicrobial resistance worldwide. During the pandemic, the extensive empirical use of antibiotics, in both hospitalized and outpatient settings, particularly broad-spectrum agents, together with prolonged hospital stays, increased intensive care unit admissions, and disruptions in antimicrobial stewardship programs, may have contributed to the acceleration of bacterial resistance [11,12]. Several studies reported important changes in the distribution and antimicrobial susceptibility profiles of Gram-negative pathogens, including *E. coli*, during and after the pandemic period [13]. In Romania, where antimicrobial resistance rates were already among the highest in Europe, national surveillance data showed a notable decrease in *E. coli* resistance percentages during the first years of the pandemic [14,15]. Nevertheless, the longer-term impact of the pandemic on resistance dynamics remains insufficiently characterized.

Beyond the resistance of the organism itself, which drives the use of ineffective empirical agents or of reserve antibiotics, the manner in which urine cultures are requested has come to be recognized as a modifiable driver of inappropriate antimicrobial use. UTIs account for a large share of hospital antibiotic prescribing, yet a substantial proportion of urine cultures are obtained from patients without urinary symptoms and therefore detect asymptomatic bacteriuria that is subsequently, and most often unnecessarily, treated. Because such low-yield culturing propagates avoidable prescribing and, in turn, the selection of resistance, urine culture ordering has become a principal target of diagnostic stewardship [16]. Meaningful surveillance of this behavior requires a denominator that reflects hospital activity rather than raw test volume, and the number of urine cultures per 1000 occupied bed-days (OBD) is now a widely used measure of testing intensity [17]. Reported utilization varies markedly with case-mix and local practice; ordering rates of the order of 40 cultures per 1000 bed-days have been described on acute wards, falling to approximately 12 per 1000 bed-days after sustained stewardship interventions [17]. OBD normalization is especially important over periods of fluctuating hospital capacity such as the COVID-19 pandemic, when a fall in the absolute number of urine cultures may merely track a contraction in admissions rather than a genuine change in clinician ordering behavior [18].

The present study aims to evaluate, in a tertiary care hospital in Romania, the evolution of both the antimicrobial resistance patterns of *E. coli* isolates and urine culture utilization, expressed per 1000 occupied bed-days, across a five-year period. By comparing resistance trends and OBD-adjusted culturing intensity across the years, this study intends to characterize the changes in *E. coli* resistance profiles over a five-year period encompassing the COVID-19 pandemic, as well as concurrent shifts in diagnostic testing behavior, and to provide updated epidemiological data that may support empirical therapeutic decisions, antimicrobial and diagnostic stewardship interventions, and infection prevention strategies.

## 2. Materials and Methods

This is a retrospective cohort study of *E. coli* isolated from adult patients’ urine cultures at the Central Military University Emergency Hospital “Dr. Carol Davila”, Bucharest, between January 2019 and December 2023. The inclusion criterion was an uncontaminated urine culture from an adult patient yielding *E. coli* with an available susceptibility result. Exclusion criteria were: patients without identifying data (name or national identification number); and duplicate isolates, only the first *E. coli* isolate per patient was retained, with subsequent isolates from the same patient within a 90-day episode excluded as duplicates. For each included isolate, the electronic patient records were reviewed and the following data were collected: age, sex, request date, isolated uropathogen, tested antimicrobial agents, susceptibility testing method, ward specialty, and urinalysis.

**Microbiological tests**

The isolates of *E. coli* were identified by matrix-assisted laser desorption ionization–time-of-flight mass spectrometry (MALDI-TOF MS) using the VITEK MS system (bioMérieux S.A., Marcy-l’Étoile, France). Antimicrobial susceptibility testing was performed using automated systems: VITEK 2 Compact (bioMérieux S.A., Marcy-l’Étoile, France).

The sensitivity thresholds for the tested antibiotics were established according to the regulations in force at the time of sample processing, namely the Clinical and Laboratory Standards Institute (CLSI) recommendations, versions 2019–2021 for the period 2019–2021, and the European Committee on Antimicrobial Susceptibility Testing (EUCAST) recommendations, versions 12.0 and 13.0 for the period 2022–2023. Two categories of isolates were defined: resistant (R) and susceptible (S and I), according to the CLSI and EUCAST recommendations.

According to EARS-Net definitions, aminopenicillin-resistant *E. coli* was defined as isolates exhibiting resistance to at least one of ampicillin or amoxicillin. Third-generation cephalosporin-resistant *E. coli* included isolates resistant to at least one of cefotaxime, ceftriaxone, or ceftazidime. Carbapenem-resistant *E. coli* was defined as isolates resistant to at least one of imipenem or meropenem. Fluoroquinolone-resistant *E. coli* included isolates resistant to at least one of ciprofloxacin, levofloxacin, or ofloxacin. Aminoglycoside-resistant *E. coli* was defined as isolates resistant to at least one of gentamicin or tobramycin (13).

**Statistical analysis**

Continuous variables are summarized as mean ± standard deviation and categorical variables as counts and percentages. For the utilization analysis, testing intensity was expressed as the number of urine cultures per 1000 occupied bed-days, with OBD serving as the denominator to adjust for fluctuating hospital activity. Temporal trends were modelled with calendar year entered as a continuous predictor and the natural logarithm of OBD included as an offset, yielding incidence rate ratios (IRR) per calendar year. Because the count data were overdispersed, department-level trends were estimated using quasi-Poisson (dispersion-corrected) regression, and group-level (“overall”) trends using negative-binomial regression with cluster-robust standard errors clustered by department to account for the correlation of repeated yearly observations within units. Cumulative change over the study period was derived from the fitted models. The two COVID-19 units (intensive care and infectious diseases), which were operational only during 2020–2022, were retained in the descriptive table but their trends were estimated over that three-year interval; the department-level trend narrative was restricted to the departments with data across all five years.

For the resistance analysis, only the first *E. coli* isolate per patient was retained. Resistance proportions are reported with 95% Wilson score confidence intervals. Temporal trends in resistance were assessed in two complementary ways: an absolute change, expressed as percentage points per year and estimated from a linear probability model with calendar year as a continuous predictor, and a relative change, expressed as the odds ratio per year from logistic regression with calendar year modelled continuously; trend estimates were unadjusted. Multidrug resistance was defined as resistance to at least one agent in at least three of nine antimicrobial classes (aminopenicillins, amoxicillin–clavulanic acid, fluoroquinolones, nitrofurantoin, fosfomycin, trimethoprim–sulfamethoxazole, third-generation cephalosporins, aminoglycosides, and carbapenems). Differences in resistance across the three hospital settings (intensive care, medical, surgical) were tested with Pearson’s chi-square test (resistant vs. susceptible/intermediate; two degrees of freedom).

All tests were two-sided, with *p* < 0.05 considered statistically significant. Analyses were performed using R version 4.5.0 (R Foundation for Statistical Computing, Vienna, Austria).

## 3. Results

**Study population**

During the study period, a total of 95,840 urine samples were processed, of which 62,467 (65.2%) originated from inpatients and 33,373 (34.8%) from outpatients (Table 1). The mean age of the study population was 60.5 ± 15.8 years, and 48,056 cultures (50.1%) were obtained from female patients. Overall, 15,619 samples (16.3%) yielded a positive urine culture.

**Overall urine culture utilization**

**Figure 1:**
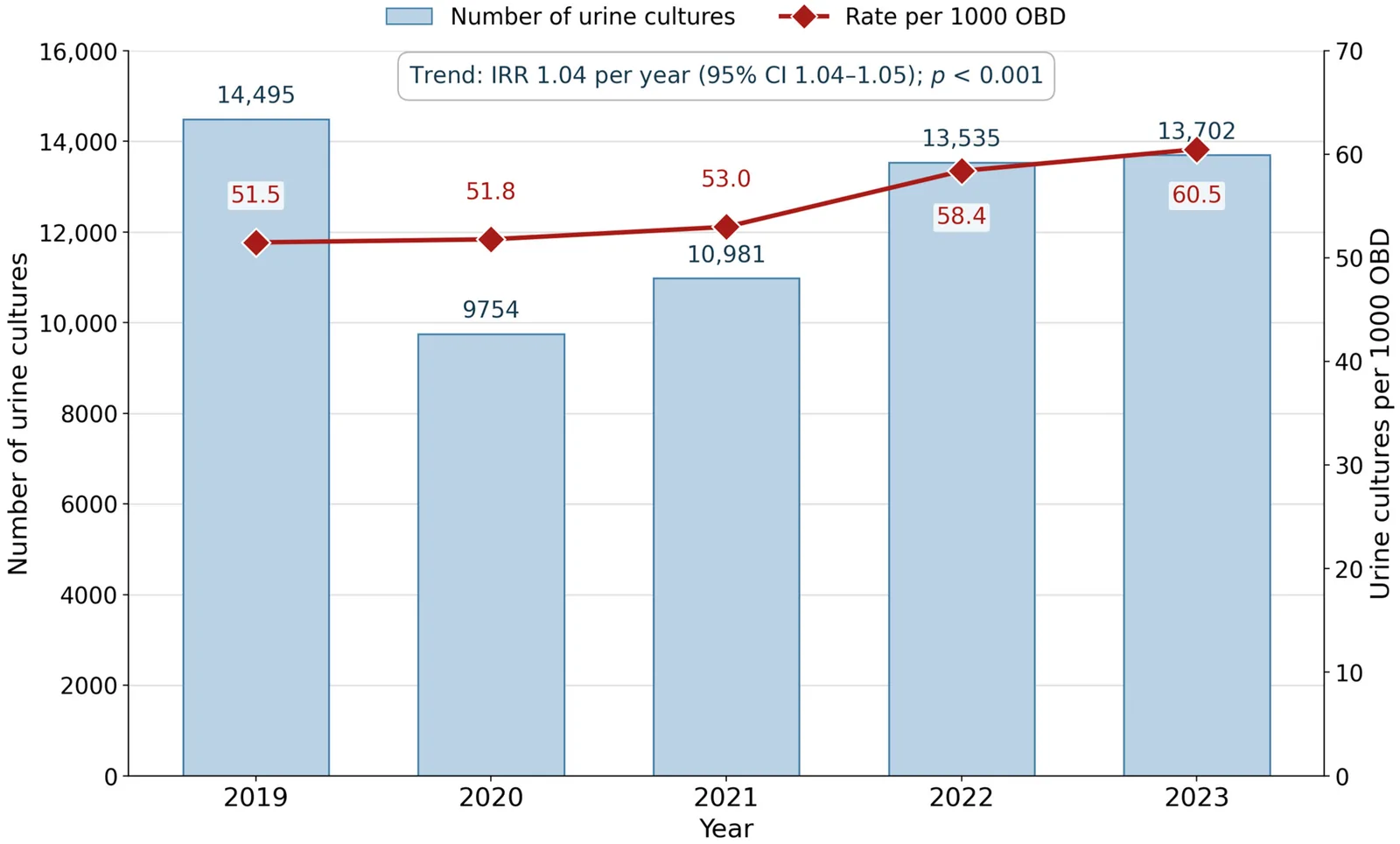
Annual urine culture samples for inpatients, 2019–2023.

Adult inpatients accounted for 62,467 urine cultures over 2019–2023. The absolute number of cultures fell by 32.7% in 2020 (14,495 to 9754), paralleling a comparable decline in OBD during the first year of the COVID-19 pandemic, before recovering to near-baseline values in 2022–2023 (Figure 1). When adjusted for OBD, however, utilization did not decline: the rate was stable across 2019–2020 (51.5 and 51.8 cultures per 1000 OBD, respectively) and subsequently rose steadily to 60.5 per 1000 OBD in 2023. This corresponded to an annual increase of 4.4% (incidence rate ratio [IRR] 1.044, 95% CI 1.039–1.050; *p* < 0.001), equivalent to a cumulative increase of 17.3% over the study period.

**Utilization trends by clinical group**

Aggregated into three clinical groups (ICU, medical and surgical departments), OBD-adjusted utilization differed substantially by care setting and diverged over time (Table 2). Medical departments were the principal driver of the institutional increase: after an initial decline in 2020 (52.5 per 1000 OBD), utilization rose progressively to 78.3 in 2023, corresponding to an average annual increase of approximately 8% (IRR 1.08, 95% CI 1.05–1.11; *p* < 0.001; cumulative change +34.3%), a trend that was consistent across the majority of medical departments. Intensive care units maintained the highest utilization throughout, peaking at 179.5 per 1000 OBD in 2020 before falling to 123.9 in 2023; this apparent reduction did not reach statistical significance (annual IRR 0.95, 95% CI 0.86–1.05; *p* = 0.31; cumulative change −18.3%). The group-level non-significance reflected heterogeneity among the four constituent units rather than an absence of change: the decline was concentrated in General ICU and the transient ICU COVID cohort, whereas Cardiac ICU and Cardiac Critical Care Unit were stable or rising (Table 2). Surgical departments exhibited the lowest utilization and remained essentially stable across the five years (annual IRR 1.01, 95% CI 0.92–1.11; *p* = 0.80; cumulative change +5.0%).

**Department-level trends**

**Figure 2:**
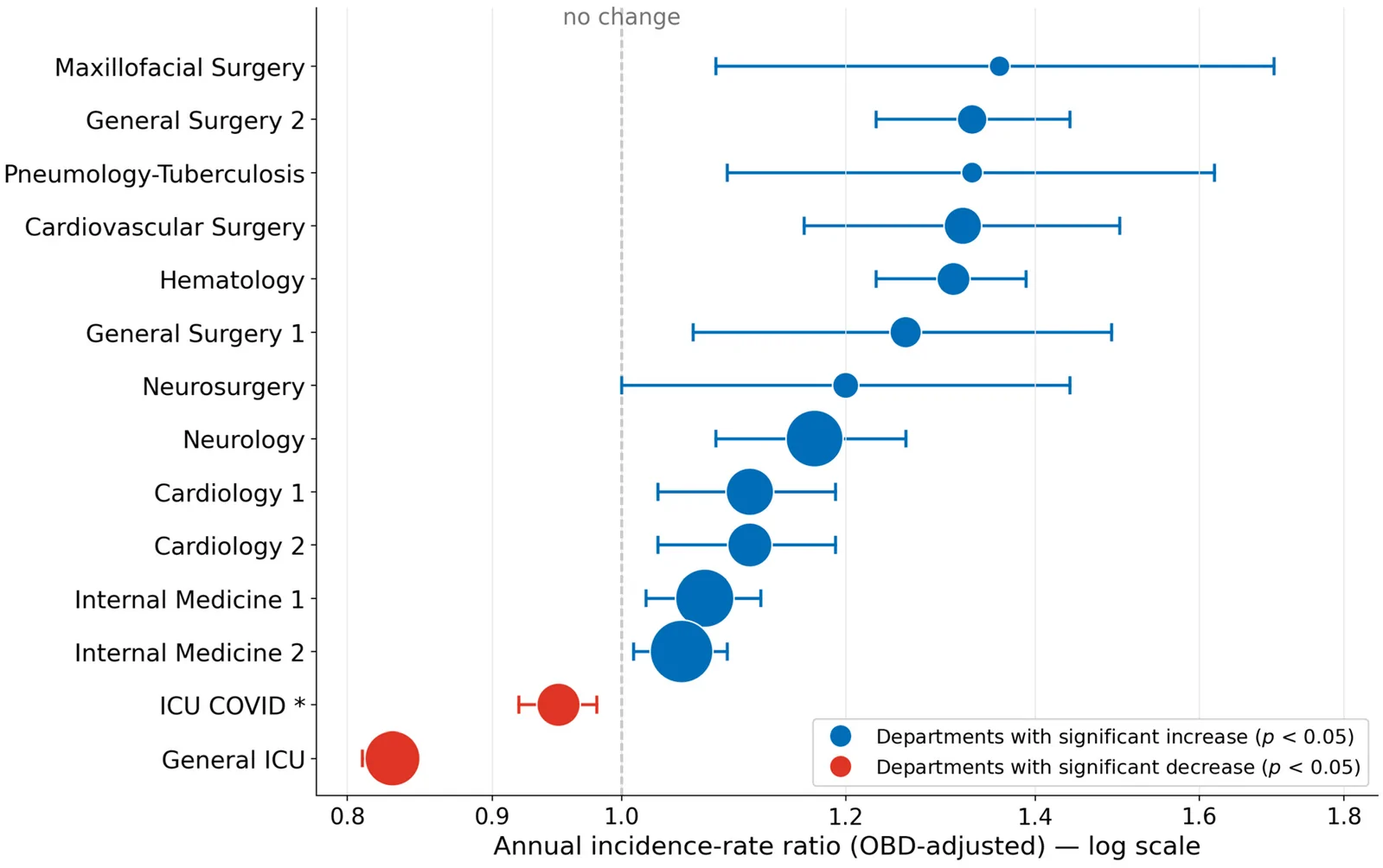
Annual trend in urine culture utilization in departments with statistically significant increase/decrease, 2019–2023. IRR > 1, increasing trend; IRR < 1, decreasing trend. The size of each circle is proportional to the total number of urine cultures processed by the department over the study period. * COVID-19 units (ICU COVID and ID COVID) were operational only during 2020–2022; their trends were estimated over these three years rather than the full 2019–2023 period.

At the department level, and restricting attention to the 32 departments with data across all five years (without the two COVID-19 units, active only in 2020–2022), 13 departments changed significantly (12 increases and 1 decrease; Table 2 and Figure 2). Significant increases spanned both medical services (including internal medicine, cardiology, neurology, hematology, and pneumology-tuberculosis) and several surgical services (general surgery, cardiovascular, maxillofacial, and neurosurgery), with annual rate ratios ranging from 1.05 to 1.36. The only significant decrease occurred in intensive care, a marked and sustained fall in General ICU (annual IRR 0.83, 95% CI 0.81–0.84; *p* < 0.001). Utilization varied more than 200-fold across departments, from below 1 to over 200 cultures per 1000 OBD, underscoring substantial between-department heterogeneity in testing practice. Among the highest-utilization departments (pooled rate >100 cultures per 1000 OBD), the institutional increase was again attributable to the medicine wards, whereas the intensive care units either declined (General ICU) or showed no significant change (Cardiac Critical Care Unit, Cardiac ICU; Table 2).

Among the processed urine samples, 15,619 yielded positive cultures, corresponding to an overall positivity rate of 16.3%. Following the exclusion of duplicate episodes, a total of 14,075 first isolates were included in the final analysis. Of these, 9199 isolates (65.4%) were obtained from female patients and 4876 (34.6%) from male patients. The mean (±SD) patient age was 65.1 (±15.1) years.

Out of 14,075 positive urine cultures, 7957 (56.5%) yielded *E. coli*, the most frequently isolated uropathogen. Most *E. coli* isolates were obtained from women (6088; 76.5%), and the mean patient age was 64.1 (±15.7) years. Patient and isolate characteristics are summarized in Table 3.

Over the study period (2019–2023), resistance to most antimicrobials remained stable, with no statistically significant temporal trend for the majority of agents. Two agents were exceptions, both showing significant declines. Nitrofurantoin resistance fell by −1.13 percentage points per year (95% CI −1.38 to −0.89; *p* < 0.001), from 5.5% in 2019 to 0.9% in 2023; this was confirmed on logistic regression (OR 0.63 per year, 95% CI 0.56–0.70; *p* < 0.001). Amoxicillin–clavulanic acid resistance likewise declined, by −1.19 percentage points per year (95% CI −1.76 to −0.62; *p* < 0.001), from 21.8% in 2019 to 17.6% in 2023 (OR 0.92 per year, 95% CI 0.89–0.96; *p* < 0.001). Fluoroquinolone resistance did not change significantly (+0.26 pp/year, 95% CI −0.38 to +0.90; *p* = 0.43), fluctuating between 22.0% and 27.9% without a consistent direction. Non-significant downward trends were observed for aminopenicillins (−0.62 pp/year; 95% CI −1.36 to +0.12; *p* = 0.10) and third-generation cephalosporins (−0.38 pp/year; 95% CI −0.88 to +0.11; *p* = 0.13), while resistance to trimethoprim–sulfamethoxazole (−0.16 pp/year; *p* = 0.65), aminoglycosides (−0.11 pp/year; *p* = 0.58), fosfomycin (+0.19 pp/year; *p* = 0.29) and carbapenems (+0.02 pp/year; *p* = 0.49) was essentially unchanged. Consistent with these findings, the burden of multidrug resistance did not increase over time (MDR: OR 0.99 per year, 95% CI 0.96–1.03; *p* = 0.62), and combined fluoroquinolone/aminoglycoside/third-generation-cephalosporin co-resistance likewise remained stable (OR 0.97 per year, 95% CI 0.89–1.05; *p* = 0.41) (Table 4).

Resistance differed significantly across hospital settings for most agents (Table 5). Isolates from medical wards consistently showed the lowest resistance, whereas those from the ICU and surgical wards were more frequently resistant. On chi-square testing, this gradient was statistically significant for aminopenicillins (58.8% ICU vs. 54.3% medical vs. 59.2% surgical; *p* = 0.002), amoxicillin–clavulanic acid (21.6% vs. 16.8% vs. 21.7%; *p* < 0.001), fluoroquinolones (27.9% vs. 22.8% vs. 31.9%; *p* < 0.001), nitrofurantoin (4.2% vs. 2.2% vs. 3.6%; *p* = 0.001), third-generation cephalosporins (18.2% vs. 11.4% vs. 17.8%; *p* < 0.001) and aminoglycosides (8.5% vs. 6.8% vs. 10.6%; *p* < 0.001). Composite phenotypes followed the same pattern, with significantly higher rates in surgical and ICU isolates for both multidrug resistance (31.8% vs. 28.4% vs. 22.9% in surgical, ICU and medical wards; *p* < 0.001) and combined fluoroquinolone/aminoglycoside/third-generation-cephalosporin co-resistance (5.9% vs. 4.4% vs. 2.7%; *p* < 0.001). No significant between-setting differences were seen for fosfomycin (*p* = 0.57) or trimethoprim–sulfamethoxazole (*p* = 0.064).

## 4. Discussion

**Urine culture utilization**

In this five-year, single-center study of urine culture utilization among adult inpatients, testing intensity adjusted for OBD increased by 4.4% per year, despite a marked, pandemic-related fall in the absolute number of cultures in 2020. This increase was not uniform: it was driven almost entirely by medical departments (+34.3% over the period), whereas intensive care and surgical utilization did not change significantly at the group level. At the department level, testing intensity varied more than 200-fold, and 12 of 32 departments with estimable trends showed a significant temporal change, the great majority of them increasing.

A central observation is the divergence between absolute test volume and OBD-adjusted utilization during the pandemic. The 32.7% decline in cultures in 2020 closely mirrored the contraction in occupied bed-days as admissions were curtailed; once this reduction in hospital activity is taken into account, testing intensity per bed-day did not fall and subsequently rose to its highest level in 2023. Analyses that report only raw test counts may therefore misinterpret pandemic-era reductions as reduced utilization, whereas normalization to bed-days shows that clinicians’ propensity to order urine cultures was maintained and then intensified. This underscores the importance of a denominator that reflects hospital activity when monitoring diagnostic utilization over periods of fluctuating capacity.

Our findings align with the growing recognition that urine cultures are frequently overused in hospitalized patients, and that culturing in the absence of urinary symptoms most often to investigate asymptomatic bacteriuria contributes to unnecessary antimicrobial prescribing and to the selection of antimicrobial resistance [1,5,19]. The relatively low overall culture positivity in our cohort (16.3%) is consistent with a substantial component of low-yield testing. A range of diagnostic stewardship interventions including conditional or reflex urine cultures contingent on an abnormal urinalysis, removal of urine cultures from admission order sets, and requiring documentation of clinical indications have been shown to reduce culturing and downstream antibiotic exposure without compromising patient safety [16,18]. The steady rise in utilization we observed suggests that such measures were either not in place or insufficient during the study period.

The concentration of the increase within medical departments most plausibly reflects the case-mix of these wards, which admit older patients with higher burdens of comorbidity, frailty, indwelling urinary catheters, and non-specific clinical presentations, all of which lower the threshold for obtaining a urine culture [19,20,21]. This pattern identifies medical wards as the highest-yield target for diagnostic stewardship in our institution. Conversely, the absence of a significant group-level trend in intensive care, despite a visually striking decline in the pooled rate, illustrates the importance of accounting for heterogeneity between units within a group. The decline was concentrated in a single general intensive care unit and in the transient COVID cohort, while the cardiac critical care and cardiac intensive care units were stable or rising; the former may reflect a local practice change such as the adoption of catheter-associated UTI diagnostic protocols or reflex culturing [16,22], whereas the latter simply reflects the wind-down of dedicated pandemic units rather than a stewardship effect. Pooled analyses that ignore this internal structure risk overstating a uniform intensive care trend.

The more than 200-fold variation in testing intensity across departments—from fewer than one to more than 200 cultures per 1000 OBD—indicates that ordering behavior is shaped as much by local practice culture as by objective clinical need, and argues for targeted, department-specific interventions rather than uniform institution-wide policies.

**Antimicrobial resistance in urinary *E. coli***

In this five-year analysis of 7957 nonduplicative urinary *E. coli* isolates from a Romanian tertiary care hospital, *E. coli* remained the dominant uropathogen (56.5% of positive cultures) and was isolated predominantly from women, consistent with the epidemiology of UTI. For the EU and EEA, EARS-Net reported the following resistance rates for invasive *E. coli* in 2023: aminopenicillins 54.7%, fluoroquinolones 24.0%, third-generation cephalosporins 16.2%, and aminoglycosides 10.9%, while carbapenem resistance remained rare (0.3%) [14]. Our hospital recorded comparable aminopenicillin (55.4%) and fluoroquinolone (24.6%) resistance, but somewhat lower third-generation cephalosporin (12.9%) and aminoglycoside (7.5%) resistance, as well as lower triple co-resistance (FQ + AG + 3GC, 3.4% versus 5.9% in the EU/EEA). These slightly more favorable figures are consistent with the national antimicrobial resistance data, and may reflect local prescribing patterns [14].

The opposing trajectories of the two principal oral first-line agents, nitrofurantoin and fosfomycin, are best interpreted in the context of methodological and supply-related factors rather than intrinsic resistance evolution. Nitrofurantoin resistance fell progressively and significantly across all five years (5.5% to 1.2%). Fosfomycin showed the mirror-image pattern, with resistance dropping modestly during the pandemic and then rising sharply to 5.4% in 2022–2023. This apparent increase, however, must be interpreted with considerable caution, because the criteria for susceptibility categorization changed during the study interval. Between 2019 and 2021, fosfomycin susceptibility was interpreted according to CLSI breakpoints, under which isolates with an MIC ≤ 64 mg/L were classified as susceptible. From 2022 onward, EUCAST criteria were progressively implemented with substantially lower thresholds (MIC ≤ 32 mg/L for intravenous fosfomycin and ≤8 mg/L for oral formulations, subsequently harmonized to ≤8 mg/L for all formulations). Because this downward shift in breakpoints mechanically reclassifies as resistant a proportion of isolates that would previously have been reported as susceptible, much of the post-pandemic rise is likely an artefact of stricter interpretive standards rather than a true change in microbial susceptibility. Two further factors compound this effect: the deregistration of fosfomycin-trometamol by its principal supplier in 2022, which prompted a shift toward extemporaneous magistral preparation, albeit with supply gaps during the initial phase, and a marked contraction in the number of isolates tested for fosfomycin in the 2022–2023 period (from 3836/4418 in 2019–2021 to 857/3539 in 2022–2023), yielding a smaller and more selected denominator. Taken together, these observations indicate that the fosfomycin trend should not be read as evidence of emerging resistance, and they illustrate how concurrent changes in breakpoint standards, drug supply and testing practice can distort surveillance signals for a single agent.

From a therapeutic standpoint, our data carry clear implications for the empirical management of community UTI. Nitrofurantoin and fosfomycin retained excellent activity, with resistance below 3% overall (2.6% and 2.8%, respectively), and nitrofurantoin resistance in fact declined further over the study period. These agents should therefore be the preferred empirical choices for lower UTI. By contrast, applying the widely used 20% threshold above which an agent is no longer recommended for empirical use, all three of aminopenicillins (55.4%), trimethoprim–sulfamethoxazole (30.3%) and fluoroquinolones (24.6%) exceed the limit and should not be relied upon for empirical therapy in this setting. Reassuringly, carbapenem resistance remained negligible throughout (0.1%) and triple co-resistance to fluoroquinolone, aminoglycoside and third-generation-cephalosporin, key agents for the treatment of severe or complicated UTI, was uncommon (3.4%), indicating that oral and parenteral options for most patients are preserved.

**Strengths and limitations**

Strengths of this study include its multi-year timeframe spanning the COVID-19 pandemic, department-level granularity across 34 clinical units, normalization to occupied bed-days to adjust for fluctuating hospital capacity and variable patient attendance, and the use of dispersion-corrected (quasi-Poisson) and cluster-robust negative-binomial models, which avoid the over-confident inference that naive Poisson regression would have produced for these overdispersed, clustered data.

For the resistance analysis, restriction to the first isolate per patient avoided duplicate-sampling bias, and susceptibility definitions followed EUCAST/EARS-Net conventions, allowing direct comparison with European surveillance data. Although the data derive from a single tertiary military hospital, this is unlikely to limit the interpretation of the resistance findings: because urinary *E. coli* infections are predominantly community-acquired and arise from widespread, persistent intestinal carriage, the isolates largely reflect the community and regional reservoir rather than a narrow hospital-selected population. Furthermore, the wide catchment area of the hospital and the centralized processing of samples in a high-throughput microbiology laboratory—with analytical standards likely exceeding those of dispersed community laboratories—represent additional strengths for the quality and consistency of the resistance data.

Several limitations should be acknowledged. First, this is a single-center analysis. This chiefly constrains the generalizability of the urine culture utilization findings, which reflect local ordering behavior, laboratory practices, and stewardship programs and may differ substantially at institutions with a different case-mix. The resistance findings are likely more transferable: susceptibility testing and reporting followed EUCAST/EARS-Net conventions, the observed *E. coli* resistance profile was broadly consistent with European surveillance data, and—given the size of the hospital and its wide catchment area, which draws patients from beyond Bucharest—these estimates are likely to approximate regional rather than purely institutional epidemiology. Nonetheless, absolute resistance rates remain influenced by local antimicrobial-use patterns and should be interpreted with corresponding caution. Second, we lacked clinical data on the indication for testing and so could not determine what proportion of cultures were appropriate, nor distinguish investigation of symptomatic UTI from asymptomatic bacteriuria.

The pandemic-period strata are defined by calendar interval rather than by individual COVID-19 status, so attribution of resistance changes to the pandemic remains ecological and associative.

A further limitation is the transition from CLSI (2019–2021) to EUCAST (2022–2023) breakpoints midway through the study. A direct comparison of the CLSI (M100, 29th/30th/31st editions) and EUCAST (v12.0/v13.0) criteria for the agents analyzed showed that this change materially altered resistance categorization only for fosfomycin, for which the two systems apply markedly different breakpoints (CLSI susceptible ≤ 64 mg/L, resistant ≥ 256 mg/L; EUCAST susceptible ≤ 8 mg/L, resistant > 8 mg/L). This effect was compounded by a limitation of the testing method for 2022–2023 period: fosfomycin susceptibility was determined from the automated broth-based MIC, and because the panel’s lowest tested concentration was 64 mg/L—adequate for the CLSI susceptible breakpoint (≤64 mg/L) but above the EUCAST breakpoint (≤8 mg/L)—isolates with MIC ≤ 64 mg/L could not be classified as susceptible once EUCAST criteria were applied, which inflated apparent resistance from 2022–2023. The same phenomenon also explains the reduced number of isolates with an interpretable fosfomycin result. Accordingly, the apparent rise in fosfomycin resistance between 2021 (0.4%) and 2022 (8.9%) coincides precisely with the switch and most likely reflects the more stringent EUCAST breakpoint rather than a true epidemiological change. For the remaining agents, the resistance boundary was either identical (third-generation cephalosporins via cefotaxime/ceftriaxone, fluoroquinolones, nitrofurantoin, trimethoprim–sulfamethoxazole) or only nominally different (aminopenicillins, gentamicin, tobramycin, ceftazidime); in the latter cases the corresponding time series showed no step change at the 2021–2022 transition, indicating that the breakpoint switch did not generate artefactual temporal trends. We did not perform genotypic characterization of ESBL or carbapenemase determinants.

## 5. Conclusions

Among adult inpatients between 2019 and 2023, urine culture utilization rose steadily after adjustment for hospital activity (annual IRR 1.044), driven predominantly by medical departments and characterized by more than 200-fold heterogeneity between clinical units; the pandemic-era fall in absolute test numbers reflected contracting bed-days rather than a genuine change in ordering behavior, which bed-day normalization made apparent. In parallel, resistance in urinary *E. coli* remained broadly stable, with a transient, pandemic-associated rise in fluoroquinolone, third-generation cephalosporin and trimethoprim–sulfamethoxazole resistance that subsequently reversed, a steady decline in nitrofurantoin resistance, and an overall profile closely aligned with, and slightly more favorable than, contemporary European surveillance data. Nitrofurantoin and fosfomycin retained excellent activity (<3% resistance) and remain the most dependable empirical options for lower UTI, contingent on stable supply, whereas aminopenicillins, trimethoprim–sulfamethoxazole and fluoroquinolones all exceeded the 20% threshold and should not be used empirically; carbapenem resistance remained negligible and triple co-resistance uncommon, so options for most patients are preserved. Taken together, these findings identify high-utilization, rising departments—particularly the medical wards—as priority targets for diagnostic stewardship, and argue for a combined strategy of diagnostic and antimicrobial stewardship, resilient supply chains for agents achieving high, selective urinary concentrations, and continued bed-day-normalized, department-level surveillance to detect trends that aggregate or volume-based monitoring would miss.

## Figures and Tables

**Table 1:** Demographic characteristics of patients with processed urine cultures, 2019–2023.

Characteristic	Value
Total urine cultures, *n*	95,840
Age, years, mean ± SD	60.5 ± 15.8
Setting, *n* (%)	
Inpatient	62,467 (65.2)
Outpatient	33,373 (34.8)
Sex, *n* (%)	
Female	48,056 (50.1)
Male	47,784 (49.9)
Culture result, *n* (%)	
Positive	15,619 (16.3)
Negative	80,221 (83.7)

**Table 2:** Annual trend in urine culture utilization by department and clinical group, 2019–2023.

Department	Urine Cultures, *n*	Rate 2019, Urine Cultures/1000 OBD	Rate 2023, Urine Cultures/1000 OBD	Annual IRR (95% CI)	*p*-Value
Intensive care					
Cardiac Critical Care Unit	5321	203.9	201.7	0.99 (0.91–1.08)	0.855
General ICU	3791	160.0	72.8	0.83 (0.81–0.84)	<0.001
Cardiac ICU	2281	87.2	114.7	1.04 (0.89–1.21)	0.647
ICU COVID *	1456	—	—	0.95 (0.92–0.98)	<0.001
Intensive care, overall	12,849	155.0	123.9	0.95 (0.86–1.05)	0.306
Medical					
Cardiology 1	2094	40.5	63.2	1.11 (1.03–1.19)	0.007
Cardiology 2	1568	27.8	43.4	1.11 (1.03–1.19)	0.009
Dermatology	368	24.2	27.4	1.03 (0.98–1.08)	0.246
Endocrinology	1318	52.0	67.2	1.04 (0.97–1.12)	0.251
Gastroenterology	5247	97.4	112.0	1.04 (0.98–1.11)	0.199
Hematology	497	11.2	31.1	1.31 (1.23–1.39)	<0.001
Infectious Diseases	6098	86.4	131.2	1.13 (0.98–1.30)	0.088
Infectious Diseases COVID *	389	—	—	1.38 (0.97–1.95)	0.073
Internal Medicine 1	4741	122.0	160.3	1.07 (1.02–1.12)	0.008
Internal Medicine 2	6334	162.6	198.7	1.05 (1.01–1.09)	0.015
Nephrology	2225	205.4	233.4	1.02 (0.88–1.18)	0.793
Neurology	4320	57.0	112.0	1.17 (1.08–1.26)	<0.001
Oncology	203	9.4	9.7	1.05 (0.93–1.20)	0.403
Pneumology-tuberculosis (TB)	83	4.2	11.9	1.33 (1.09–1.62)	0.005
Psychiatry	1668	32.9	28.9	0.97 (0.90–1.05)	0.477
Pulmonology	968	22.3	41.5	1.20 (0.99–1.45)	0.063
Rehabilitation 1	610	15.9	16.9	1.04 (0.92–1.17)	0.550
Rehabilitation 2	722	18.3	22.1	1.05 (0.99–1.10)	0.086
Medical, overall	39,453	61.2	78.3	1.08 (1.05–1.11)	<0.001
Surgical					
Cardiovascular Surgery	814	17.2	43.6	1.32 (1.16–1.50)	<0.001
ENT	62	3.0	2.8	1.02 (0.90–1.16)	0.756
General Surgery 1	406	3.1	8.4	1.26 (1.06–1.49)	0.008
General Surgery 2	325	3.7	11.1	1.33 (1.23–1.44)	<0.001
Gynecology	140	4.0	5.2	1.09 (0.93–1.27)	0.283
Maxillofacial Surgery	77	1.4	5.3	1.36 (1.08–1.70)	0.008
Neurosurgery	193	2.3	3.6	1.20 (1.00–1.44)	0.046
Ophthalmology	25	1.0	0.8	1.00 (0.86–1.17)	0.977
Orthopedics	1307	47.8	41.8	1.00 (0.91–1.09)	0.940
Plastic Surgery	75	7.9	1.9	0.82 (0.51–1.34)	0.435
Thoracic Surgery	80	1.3	1.7	1.10 (0.94–1.30)	0.230
Urology	6661	179.5	145.2	0.95 (0.87–1.04)	0.297
Surgical, overall	10,165	24.0	24.1	1.01 (0.92–1.11)	0.795

IRR, incidence rate ratio (per calendar year); OBD, occupied bed-days; ENT, Ear-Nose-Throat. Rates are expressed per 1000 OBD. Department-level IRRs were estimated by quasi-Poisson regression (dispersion-corrected) with a log-OBD offset; group-level (“overall”) IRRs by negative-binomial regression with cluster-robust standard errors by department. * COVID-19 unit (ICU COVID and ID COVID) were active only in 2020–2022; their trends were estimated over these 3 years, not the full 2019–2023 period; in red departments with significant decrease, in blue departments with significant increase.

**Table 3:** Characteristics of patients with *E. coli*–positive urine cultures, 2019–2023.

Characteristic	Value
*E. coli* isolates, *n* Sex, *n* (%)	7957
Female	6088 (76.5)
Male	1869 (23.5)
Age, years—mean (±SD)	64.1 (±15.7)
*E. coli* isolates by hospital setting, n (%)	
ICU	610 (7.7)
Medical	6101 (76.7)
Surgical	1246 (15.6)

**Table 4:** Antimicrobial resistance among first-isolate urinary *E. coli* (2019–2023): overall temporal trends and year-by-year resistance.

Antimicrobial Class/Phenotype	*n*	2019 % (95% CI)	2020 % (95% CI)	2021 % (95% CI)	2022 % (95% CI)	2023 % (95% CI)	Yearly Change, pp/yr (95% CI; *p*)	OR per Year (95% CI; *p*)
Aminopenicillins	7807	55.9 (53.6–58.1)	58.6 (55.5–61.6)	55.0 (52.3–57.6)	54.3 (51.9–56.6)	54.3 (52.0–56.7)	−0.62 (−1.36 to +0.12; 0.10)	0.98 (0.95–1.00; 0.10)
**Amoxicillin** **–clavulanic acid**	**7763**	**21.8** **(20.0–23.7)**	**19.2** **(16.9–21.7)**	**14.6** **(12.9–16.6)**	**16.0** **(14.4–17.8)**	**17.6** **(15.9–19.5)**	**−1.19** **(−1.76 to −0.62; <0.001)**	**0.92** **(0.89–0.96; <0.001)**
Fluoroquinolones	7953	22.0 (20.2–23.9)	26.2 (23.7–28.8)	27.9 (25.6–30.4)	25.4 (23.4–27.5)	23.1 (21.2–25.2)	+0.26 (−0.38 to +0.90; 0.43)	1.01 (0.98–1.05; 0.43)
**Nitrofurantoin**	**7482**	**5.5** **(4.5–6.7)**	**3.8** **(2.8–5.1)**	**1.7** **(1.2–2.6)**	**1.5** **(1.0–2.2)**	**0.9** **(0.6–1.5)**	**−1.13** **(−1.38 to −0.89; <0.001)**	**0.63** **(0.56–0.70; <0.001)**
Fosfomycin	4693 *	2.8 (2.1–3.6)	4.6 (3.3–6.5)	0.4 (0.2–1.0)	8.9 (6.1–12.7)	3.5 (2.3–5.4)	+0.19 (−0.16 to +0.53; 0.29)	1.07 (0.94–1.21; 0.29)
Trimethoprim–sulfamethoxazole	7926	28.4 (26.5–30.5)	32.9 (30.2–35.7)	32.9 (30.4–35.4)	30.6 (28.5–32.8)	28.1 (26.1–30.3)	−0.16 (−0.84 to +0.52; 0.65)	0.99 (0.96–1.03; 0.65)
Third-generation cephalosporins	7957	12.1 (10.7–13.6)	16.4 (14.3–18.7)	13.9 (12.2–15.9)	12.2 (10.8–13.8)	11.6 (10.1–13.1)	−0.38 (−0.88 to +0.11; 0.13)	0.97 (0.92–1.01; 0.13)
Aminoglycosides	7930	7.5 (6.4–8.7)	8.4 (6.9–10.2)	7.7 (6.4–9.3)	6.9 (5.8–8.2)	7.5 (6.4–8.8)	−0.11 (−0.50 to +0.28; 0.58)	0.98 (0.93–1.04; 0.58)
Carbapenems	7955	0.0 (0.0–0.2)	0.2 (0.0–0.7)	0.1 (0.0–0.4)	0.2 (0.1–0.5)	0.1 (0.0–0.3)	+0.02 (−0.03 to +0.06; 0.49)	1.20 (0.71–2.01; 0.49)
Multidrug resistance (≥3 classes)	7957	24.5 (22.6–26.4)	25.2 (22.7–27.8)	25.8 (23.6–28.2)	25.0 (23.1–27.1)	23.6 (21.7–25.7)	−0.16 (−0.80 to +0.48; 0.62)	0.99 (0.96–1.03; 0.62)
FQ + AG + 3GC co-resistance	7929	2.9 (2.3–3.8)	5.0 (3.8–6.4)	3.7 (2.8–4.8)	2.9 (2.2–3.7)	3.1 (2.4–4.0)	−0.11 (−0.38 to +0.15; 0.41)	0.97 (0.89–1.05; 0.41)

Data are % resistant (95% CI, Wilson score). Resistance = R; intermediate (I) grouped with susceptible. n = isolates with a valid result for the class. * 3836 of 4418 isolates (86.8%) were tested in 2019–2021, 857 of 3539 isolates (24.2%) were tested in 2022–2023. Fluoroquinolones: ciprofloxacin/levofloxacin/ofloxacin. Third-generation cephalosporins: cefotax-ime/ceftriaxone/ceftazidime. Aminoglycosides: gentamicin/tobramycin. Carbapenems: meropenem/imipenem. Multidrug resistance (MDR)—resistance to ≥1 agent in ≥3 of the following 9 classes: aminopenicillins, amoxicillin–clavulanic acid, fluoroquinolones, nitrofurantoin, fosfomycin, trimethoprim–sulfamethoxazole, third-generation cephalosporins, aminoglycosides, and carbapenems. Yearly change (pp/yr) from a linear probability model; pp = percentage points. OR per year from logistic regression (calendar year modelled as a continuous variable). Trend estimates are unadjusted.

**Table 5:** Resistance in first-isolate urinary *E. coli* by hospital setting, with tests of association (2019–2023).

Antimicrobial Class/Phenotype	*n*	Overall % R	ICU % R	Medical % R	Surgical % R	*p*-Value (χ^2^), ICU vs. Medical vs. Surgical
Aminopenicillins	7807	55.4	58.8	54.3	59.2	**0.002**
Amoxicillin–clavulanic acid	7763	18.0	21.6	16.8	21.7	**<0.001**
Fluoroquinolones	7953	24.6	27.9	22.8	31.9	**<0.001**
Nitrofurantoin	7482	2.6	4.2	2.2	3.6	**0.001**
Fosfomycin	4693 *	2.8	3.5	2.7	3.1	0.57
Trimethoprim–sulfamethoxazole	7926	30.3	32.2	29.6	32.6	0.064
Third-generation cephalosporins	7957	12.9	18.2	11.4	17.8	**<0.001**
Aminoglycosides	7930	7.5	8.5	6.8	10.6	**<0.001**
Carbapenems	7955	0.1	0.2	0.1	0.0	NA
Multidrug resistance (≥3 classes)	7957	24.7	28.4	22.9	31.8	**<0.001**
FQ + AG + 3GC co-resistance	7929	3.4	4.4	2.7	5.9	**<0.001**

Values are the percentage of tested isolates resistant (R) in each setting. *p*-values are from Pearson’s chi-square test comparing resistance (R vs. susceptible/intermediate) across the three settings—ICU, medical and surgical—(2 degrees of freedom). * 3836 of 4418 isolates (86.8%) were tested in 2019–2021, 857 of 3539 isolates (24.2%) were tested in 2022–2023. NA, not aplicable

## Data Availability

The data presented in this study are available on request from the corresponding author.

## Abbreviations

The following abbreviations are used in this manuscript:

AMR	Antimicrobial resistance
UTI	Urinary tract infection
EARS-Net	European Antimicrobial Resistance Surveillance Network
EU	European Union
EEA	European Economic Area
ESBL	Extended-spectrum β-lactamase
OBD	Occupied bed-days
MALDI-TOF MS	Matrix-assisted laser desorption ionization–time-of-flight mass spectrometry
CLSI	Clinical and Laboratory Standards Institute
EUCAST	European Committee on Antimicrobial Susceptibility Testing
IRR	Incidence rate ratios
ICU	Intensive care unit
ENT	Otolaryngology
MDR	Multidrug resistance
AG	Aminoglycosides
3GC	Third-generation cephalosporins
FQ	Fluoroquinolones
CARMIAAM-ROM	Antibiotic consumption, microbial resistance, and healthcare-associated infections in Romania
